# Socioeconomic inequalities, health damaging behavior, and self-perceived health in Serbia: a cross-sectional study

**DOI:** 10.3325/cmj.2012.53.254

**Published:** 2012-06

**Authors:** Janko Janković, Teresa Janević, Olaf von dem Knesebeck

**Affiliations:** 1 Institute of Social Medicine, Faculty of Medicine, University of Belgrade, Belgrade, Serbia.; 2 Jackson Institute for Global Affairs, Global Health Initiative, Yale University, New Haven, Conn, USA; 3 Department of Medical Sociology and Health Economics, University Medical Center Hamburg-Eppendorf, Hamburg, Germany

## Abstract

**Aim:**

To analyze the association of socioeconomic factors with self-perceived health in Serbia and examine whether this association can be partly explained by health behavior variables.

**Methods:**

We used data from the 2007 Living Standards Measurement Study for Serbia. A representative sample of 13 831 persons aged ≥20 years was interviewed. The associations between demographic factors (age, sex, marital status, and type of settlement), socioeconomic factors (education, employment status, and household consumption tertiles), and health behavior variables (smoking, alcohol consumption) and self-perceived health were examined using logistic regression analyses.

**Results:**

A stepwise gradient was found between education and self-perceived health for the total sample, men, and women. Compared to people with high education, people with low education had a 4.5 times higher chance of assessing their health as poor. Unemployed (odds ratio [OR], 1.64; 95% confidence interval [CI], 1.29-2.10), inactive (OR, 2.82; 95% CI, 2.49-3.19), and the most deprived respondents (OR, 1.17; 95% CI, 1.02-1.34) were more likely to report poor self-perceived health than employed persons and the most affluent group. After adjustment for demographic and health behavior variables, the magnitudes of all associations decreased but remained clearly and significantly graded.

**Conclusions:**

This study revealed inequalities in self-perceived health by socioeconomic position, in particular educational and employment status. The reduction of such inequalities through wisely tailored interventions that benefit people’s health should be a target of a national health policy in Serbia.

Socioeconomic inequalities in health between and within countries have received considerable attention in health research. There are many measures that are of potential importance to the study of inequalities in health, but self-perceived health has been a very good source of information on subjective health status, incorporating aspects of both physical and mental health (1). This measure, based on a single-item, has been recommended as a population health measure by the World Health Organization and European Union Commission (2,3). The shape of health inequalities typically follows an inverse gradient, ie, the lower the socioeconomic status, the higher the probability of reporting a poor self-perceived health (4,5). Individuals with lower educational attainment or income, unemployed individuals, and individuals employed in manual occupations, are more likely to have poor self-perceived health (6-8). However, despite this typical pattern, the magnitude of socioeconomic inequalities in health varies widely in different populations (9).

A consistent association between socioeconomic determinants and health related variables has been found in many European countries (10-12). Carlson demonstrated that the so-called European health divide, documented for mortality, was also noticeable in self-perceived health (13). Mackenbach et al compared the magnitude of inequalities in self-assessed health among 22 countries in Europe and found that in almost all countries the rates of poorer self-assessments of health were substantially higher in groups of lower socioeconomic status, while the magnitude of the inequalities between groups of higher and lower socioeconomic status was much larger in some countries than in others (14). On average, people from Eastern European countries rated their health worse than those from Western European countries (13,14). Poor health status in Eastern Europe may be influenced by unhealthy lifestyles associated with lack of information about health and behavior (15). East-west difference in health status may be partly explained by differences in health behaviors (such as smoking and alcohol consumption) and psychosocial factors (16).

Despite the growing literature on this issue in central and west Europe, few studies have examined the impact of socioeconomic inequalities in self-rated health in southeastern Europe. In Serbia, a country still in the process of transition, little is known about health inequalities (17,18). Several recently published studies have brought to light the significance of socioeconomic inequalities in Serbia: in the prevalence of chronic diseases (19), morbidity status (20), and smoking habits of the population (21).

The aim of this study was to analyze the association of socioeconomic factors with self-perceived health in Serbia using the 2007 Living Standards Measurement Study data (LSMS). Additionally, we examined whether this association can be partly explained by health behavior variables.

## Methods

### Study population and sample

Analyses are based on the 2007 LSMS for the Republic of Serbia residents (excluding data on Kosovo and Metohia), which was carried out by the Statistical Office of the Republic of Serbia with the financial and professional support of the Department for International Development and the World Bank (22).

A stratified three-stage sample was used. First stage units were enumeration districts, second stage units were inhabited dwellings, while third stage units were households. The sampling frame for the LSMS was based on the enumeration districts delineated for the 2002 Serbia Census, excluding those with fewer than 20 households. Enumeration districts were stratified according to six geographical regions of Serbia (Vojvodina, Belgrade, West Serbia, Šumadija and Pomoravlje, East Serbia, and South-East Serbia) and the type of settlement (urban and rural).

Out of 7140 households randomly selected for the representative sample in the Republic of Serbia, 5557 were interviewed during May and June 2007. In the interviewed households, 17 375 individuals were identified, 13 831 of whom were adults older than 20 years (6640 men and 7191 women). The final response rate in the survey was 77.8%.

Cross-sectional data were weighted to represent the Serbian population in 2002. The weights were adjusted by population projections for 2006 based on the vital statistics (birth and death rate). More details concerning sampling and weighting are available elsewhere (22).

### Instruments

Information on demographic, socioeconomic, health behavior variables, and self-perceived health was obtained through two methods of interviewing: a face-to-face interview and self-completion consumption diary. All modules of the household questionnaire, with the exception of the consumption diary, were filled in by a trained interviewer together with the respondent. The diary was left in the household and filled in by the household member in charge of daily purchases.

### Variables

Based on a literature review, the following independent variables were selected from the database: age (categorized into ten-year age-groups: 20-29 years/30-39 years etc.), sex, marital status (married or living with the partner/not married, divorced, or widowed), and type of settlement (urban/rural). Two health behavior variables were used: smoking and alcohol consumption. Smoking was dichotomized into 1 – non-smokers and 2 – current smokers (daily and occasional smokers). Those who reported alcohol intake 1-3 times per week or every day were considered to have frequent alcohol consumption and those who reported alcohol intake at least several times a year or 2-3 times per month were considered to have moderate consumption. Non-drinkers were taken as a reference value.

The variables reflecting socioeconomic position in this study originated from the LSMS database (22) and were education –defined as 1 – high (college and university degree), 2 – middle (3 or 4 years of secondary school), and 3 – low (no education, incomplete primary school and primary school); employment status categorized as 1 – employed, 2 – unemployed, and 3 – inactive (pensioners, people attending some form of education, housewives, and persons who are inactive due to family reasons, people who are ill, unable to work or elderly, and other inactive categories); and the household consumption tertiles (three groups). Two basic components of the household consumption are the value of food expenditure and the value of non-food expenditure. Household consumption was based on the UN classification of individual consumption according to purpose (COICOP classification) (23) and includes the following expenditure categories: food and non-alcoholic beverages; alcoholic beverages and tobacco; clothing and footwear; housing; furnishings, household equipment, and maintenance; health; transport; communication; recreation and culture; education; restaurants and hotels; and miscellaneous goods and services. Respondents were classified into five socio-economic groups or quintiles with the same number of individuals in each: the richest class, richer class, middle class, poorer class, and the poorest class. For the purpose of the analysis, we transformed household consumption quintiles into tertiles, which were categorized as: 1 – the most affluent group (richest class and richer class), 2 – middle class group, and 3 – the most deprived group (poorer class and poorest class). Detailed information about the household consumption can be found elsewhere (22).

A self-perceived health was used as the dependent variable and measured through a single question: “How is your health in general?” Available responses were: “very good,” “good,” “fair,” “poor,” and “very poor”. For the analysis an outcome variable was grouped into three categories: good (very good or good), fair, and poor (poor or very poor).

### Statistical analysis

Frequencies of self-perceived health by study characteristics were examined and χ^2^ statistics calculated. In order to estimate the association of socioeconomic determinants with self-perceived health, bivariate and multivariate logistic regression models were used. Self perceived health as the dependent variable was transformed into three dummy variables (fair vs good, poor vs good, and poor vs fair self-perceived health). All independent variables, whether or not statistically significant in bivariate models, were included in the multivariate logistic regression models.

To analyze whether the association between socioeconomic factors and self-perceived health can be partly explained by smoking and alcohol consumption (health behavior variables), two regression models were calculated. In the first model, we calculated a demographic adjusted odds ratio (OR) for the relation between socioeconomic factors and self-perceived health. We then introduced health behavior variables into the model and compared demographic-adjusted with demographic and lifestyle-adjusted results by quantifying the percentage change in ORs (24). Changes were calculated by using the following equation [(OR demographic variables adjusted – OR demographic and health behavior variables adjusted)/(OR demographic variables adjusted – 1)]*100. These percentage changes are used in social epidemiology to analyze and illustrate the effect of explanatory factors like health behavior on health inequalities (25,26). All analyses were repeated by sex to investigate the differences in the association between socioeconomic variables and self-perceived health in men and women. Twelve multivariate logistic regression analyses were presented, 6 when the dependent variable was fair vs good self-perceived health and 6 when the dependent variable was poor vs good self-perceived health. As an indicator of goodness of fit of the regression models, the Nagelkerke R^2^ index was used. The probability, *P* < 0.05, was taken as the minimum level of significance. All the statistical analyses were performed with the SPSS, 17.0 statistical package (SPSS Inc., Chicago, IL, USA).

## Results

In total, 13 831 adults older than 20 years (age range 20 − 100; mean age 49.47 ± 17.32) were interviewed. There were slightly more women (7191, 52.0%) than men (6640, 48.0%). Women were more prevalent in all age groups, except in the youngest. The highest percentage of respondents was found in the 50-59 age groups, both in men (21.2%) and women (20.7%). More than two-thirds of men (68.1%) were married or living with a partner, while this rate was slightly lower in women (63.1%). The majority of respondents (44.8%) had middle education and only 17.8% had high education. More than half of individuals were urban dwellers (52.0%). The unemployment rate was 12.5%.

In both sexes, a greater proportion of younger individuals, urban dwellers, more educated, and more affluent than of older, rural residents, less educated, and more deprived respondents rated their health as good (Table 1).

**Table 1:** Distribution of individuals’ self-perceived health according to demographic, socioeconomic, and health behavior variables, Serbia, 2007

Variables	Self-perceived health							
	men (n = 6640)				women (n = 7191)			
	total	good (%)	fair (%)	poor (%)	total	good (%)	fair (%)	poor (%)
Age (years):								
20-29	1179	91.8	5.7	2.5	1084	91.8	6.0	2.2
30-39	1073	82.5	12.2	5.3	1097	76.8	16.2	6.9
40-49	1189	60.5	27.4	12.1	1222	56.5	28.9	14.6
50-59	1405	41.7	33.9	24.4	1491	31.0	41.4	27.6
60-69	906	27.0	38.3	34.7	1025	14.8	35.2	50.0
70+	888	14.9	30.3	54.8	1272	9.6	27.5	62.9
Marital status:								
married or living with a partner	4521	48.6	28.7	22.8	4537	47.1	28.4	24.4
not married, divorced or widowed	2119	68.6	15.1	16.3	2654	42.4	23.9	33.7
Type of settlement:								
urban	3365	58.1	25.5	14.4	3828	49.3	27.8	22.8
rural	3275	51.7	23.2	25.1	3363	40.9	25.5	33.6
Education:								
low	1982	36.4	26.3	37.2	3183	23.4	29.5	47.2
middle	3492	61.9	23.2	14.9	2706	60.8	25.7	13.5
high	1166	65.5	24.4	10.0	1302	67.3	22.3	10.4
Household consumption tertiles:								
the most deprived group	2871	50.3	23.7	26.1	3150	37.8	26.1	36.1
middle class group	1366	56.1	24.1	19.8	1465	47.1	27.6	25.3
the most affluent group	2403	59.9	25.3	14.9	2576	53.6	27.1	19.3
Employment status:								
employed	4018	65.1	23.7	11.2	2972	60.1	26.4	13.5
unemployed	470	72.1	17.9	10.0	525	68.6	21.1	10.3
inactive	2152	32.2	27.0	40.8	3694	30.3	27.9	41.9
Smoking:								
non-smoker	3676	52.0	24.7	23.3	5190	42.6	26.6	30.8
smoker	2873	58.9	23.8	17.3	2001	52.7	27.2	20.1
Alcohol consumption:								
no	2253	47.1	21.0	31.9	5408	41.3	26.5	32.2
moderate	2684	68.0	24.2	14.9	1527	58.0	27.4	14.6
frequent	1703	56.0	28.9	15.0	256	55.5	28.9	15.6

The highest percentage of smokers (58.9% of men and 52.7% of women) and the highest percentage of frequent alcohol consumers (56.0% of men and 55.5% of women) rated their health as good (Table 1).

In order to estimate the independent effects of socioeconomic determinants on fair vs good self-perceived health, multivariate logistic regression analyses were performed (Table 2). There was no significant association between household consumption tertiles and the fair vs good self-perceived health in the total sample, men, and women before and after adjustment for health behavior variables. The opposite was true for education. After adjustment for demographic variables, the odds of fair vs good health were higher among those with lower education (OR, 1.56; 95% CI, 1.37-1.78 for middle education and OR, 1.91; 95% CI, 1.64-2.23 for low education) (Table 2). Odds ratios in women (OR, 1.66; 95% CI, 1.38-2.00 for middle education and OR, 1.97; 95% CI, 1.59-2.44 for low education) were higher than in men (OR, 1.50; 95% CI, 1.25-1.81 for middle education and OR, 1.68; 95% CI, 1.34-2.11 for low education). After additional adjustment for health behavior variables, these associations were only slightly decreased. The biggest reduction of 8% was found in the association between low education and fair self-perceived health in the total sample. Regarding employment status, unemployed persons (OR, 1.21; 95% CI, 1.01-1.46) and inactive people (OR, 1.16; 95% CI, 1.04-1.23) were more likely to rate their health as fair than employed persons, ie, they perceived their health to be significantly worse than those of employed persons. When health behavior variables were introduced into the model, a reduction of 19% was obtained for inactive people and 29% for the unemployed. Moreover, control for health behavior variables led to a loss of significance among unemployed people.

**Table 2:** Association of socioeconomic variables with fair vs good self-perceived health in the total sample, men, and women, odds ratios and 95% confidence intervals

Variables	Fair vs good self-perceived health								
	adjusted for demographic variables*			adjusted for demographic variables* and health behavior variables^†^			change (%)^‡^		
	total	men	women	total	men	women	total	men	women
Education:									
low	1.91 (1.64-2.23)	1.68 (1.34-2.11)	1.97 (1.59-2.44)	1.84 (1.58-2.15)	1.65 (1.32-2.07)	1.95 (1.57-2.41)	-8	-4	-2
middle	1.56 (1.37-1.78)	1.50 (1.25-1.81)	1.66 (1.38-2.00)	1.53 (1.34-1.74)	1.48 (1.23-1.78)	1.63 (1.35-1.96)	-5	-4	-5
high	1 (Ref)	1 (Ref)	1 (Ref)	1 (Ref)	1 (Ref)	1 (Ref)			
Household consumption tertiles:									
most deprived	0.91 (0.81-1.01)	0.87 (0.74-1.02)	0.99 (0.84-1.16)	0.91 (0.81-1.01)	0.87 (0.74-1.02)	0.99 (0.84-1.17)	0	0	0
middle class	0.94 (0.83-1.07)	0.89 (0.74-1.07)	1.00 (0.84-1.20)	0.94 (0.82-1.07)	0.89 (0.74-1.07)	1.00 (0.84-1.20)	0	0	0
most affluent	1 (Ref)	1 (Ref)	1 (Ref)	1 (Ref)	1 (Ref)	1 (Ref)			
Employment status:									
inactive	1.16 (1.04-1.23)	1.14 (0.96-1.37)	1.04 (0.90-1.21)	1.13 (1.01-1.27)	1.15 (0.96-1.38)	1.05 (0.91-1.22)	-19	+7	+25
unemployed	1.21 (1.01-1.46)	1.25 (0.95-1.64)	1.13 (0.88-1.45)	1.15 (0.96-1.39)	1.22 (0.93-1.61)	1.08 (0.84-1.39)	-29	-12	-38
employed	1 (Ref)	1 (Ref)	1 (Ref)	1 (Ref)	1 (Ref)	1 (Ref)			

*Age, marital status and type of settlement.

†Smoking and alcohol consumption.

‡Changes were calculated by using [(OR demographic variables adjusted – OR demographic and health behavior variables adjusted)/(OR demographic variables adjusted – 1)] *100.

Associations between socioeconomic factors and poor vs good self-perceived health were stronger and reductions after control of health behavior variables were larger (Table 3).

**Table 3:** Association of socioeconomic variables with poor vs good self-perceived health in the total sample, men, and women, odds ratios and 95% confidence intervals

Variables	Poor vs good self-perceived health								
	adjusted for demographic variables*			adjusted for demographic variables* and health behavior variables^†^			change (%)^‡^		
	total	men	women	total	men	women	total	men	women
Education:									
low	4.49 (3.68-5.47)	4.66 (3.46-6.27)	4.18 (3.19-5.49)	3.91 (3.20-4.78)	4.46 (3.29-6.04)	3.78 (2.87-4.98)	-17	-5	-13
middle	2.28 (1.89-2.76)	2.69 (2.05-3.54)	1.99 (1.53-2.59)	2.10 (1.74-2.54)	2.49 (1.89-3.29)	1.80 (1.38-2.34)	-14	-12	-19
high	1 (Ref)	1 (Ref)	1 (Ref)	1 (Ref)	1 (Ref)	1 (Ref)			
Household consumption tertiles:									
most deprived	1.17 (1.02-1.34)	1.12 (0.92-1.37)	1.24 (1.03-1.51)	1.17 (1.01-1.34)	1.09 (0.89-1.34)	1.21 (1.01-1.48)	0	-25	-13
middle class	1.00 (0.85-1.18)	1.07 (0.85-1.35)	0.95 (0.76-1.19)	0.99 (0.84-1.16)	1.05 (0.82-1.33)	0.92 (0.73-1.15)	/	-29	-60
most affluent	1 (Ref)	1 (Ref)	1 (Ref)	1 (Ref)	1 (Ref)	1 (Ref)			
Employment status:									
inactive	2.82 (2.49-3.19)	3.69 (3.05-4.46)	2.14 (1.79-2.55)	2.49 (2.19-2.84)	3.39 (2.79-4.12)	2.08 (1.74-2.48)	-18	-11	-5
unemployed	1.64 (1.29-2.10)	1.74 (1.22-2.47)	1.52 (1.08-2.14)	1.45 (1.13-1.86)	1.56 (1.09-2.24)	1.42 (1.01-2.01)	-30	-24	-19
employed	1 (Ref)	1 (Ref)	1 (Ref)	1 (Ref)	1 (Ref)	1 (Ref)			

*Age, marital status, and type of settlement.

†Smoking and alcohol consumption. ·

‡Changes were calculated by using [(OR demographic variables adjusted – OR demographic and health behavior variables adjusted)/(OR demographic variables adjusted – 1)] *100.

Persons in the most deprived group were more likely to rate their health as poor than those in the most affluent group. Addition of health behavior variables attenuated the association only among women (13%). A stepwise gradient was found between education and poor vs good self-perceived health for the total sample, men, and women, both before and after adjustment for health behavior variables. In the first model, the OR for women with middle education was almost 2 times higher (OR, 1.99; 95% CI, 1.53-2.59) and for those with low education more than 4 times higher (OR, 4.18; 95% CI, 3.19-5.49) than in highly educated women. The gradient was more pronounced in men (OR, 2.69; 95% CI, 2.05-3.54 for middle education and OR, 4.66; 95% CI, 3.46-6.27 for low education). Addition of the health behavior variables attenuated all associations between education and poor health. The highest reduction was observed among middle educated women (19%) and low educated respondents (17%) (Table 3). Regarding employment status, inactive men and women more often had poor self-perceived health than employed men and women, both before and after adjustment for health behavior variables. Associations of similar magnitude were found among unemployed people, for which the biggest reductions in the associations due to health behavior variables were obtained (30% for total, 24% for men and 19% for women).

Тhe Nagelkerke R^2^, calculated for every logistic regression model, ranged from 0.51 to 0.61 when dependent variable was poor vs good self-perceived health and from 0.28 to 0.34 when dependent variable was fair vs good self-perceived health.

## Discussion

This cross-sectional study was the first ever to analyze the associations between socioeconomic factors and self-perceived health in Serbia. We also investigated the role of health behavior variables for the explanation of socioeconomic inequalities in self-perceived health after having adjusted for demographic variables.

Our findings revealed inequalities in self-perceived health by socioeconomic position, in particular educational and employment status. The associations of the greatest magnitude were found for educational status. Compared to people with high education, people with low education had a 4.5 times higher chance of assessing their health as poor after adjustment for demographic variables. After additional control for health behavior variables, the magnitude of the association between low education and self-perceived health decreased but remained clearly and significantly graded. The same pattern applied for people with a middle level of education. Our findings are consistent with the results of many other studies conducted in Europe (4,11,27,28), where lower educational level was associated with poorer self-perceived health. In the Baltic states (27), people with primary education were more than twice as likely to report poor health as those with tertiary education. Knesebeck and Geyer (24) analyzed the association between education and self-rated health in 22 European countries and found that men and women with high education had elevated probabilities of reporting good or very good health in most countries. These educational inequalities in health may be attributable to the fact that higher education provides more coping skills for daily life issues that could negatively affect health (within the family, social, and work environment) and offers more opportunities to solve them (20).

There are two major ways in which unemployment affects health: lack of income and ability to meet daily needs and emotional stress related to the meaning of the work, uncertain future, loss of self-esteem, and identity (29). In the present study, employment status was strongly associated with self-perceived health, but less so than educational level. Unemployed and inactive persons were more likely to report poor self-perceived health than employed persons. Adding health behavior variables into the model attenuated the association between employment status and self-perceived health, but the association remained highly significant. A study conducted in Sweden (30) found that those who retired early and were unemployed were more likely to have poor self rated health than employed participants. According to the authors, economic hardship affects these groups in particular. Poor self-perceived health was more frequent among unemployed than among employed people in Estonia and Finland as well (31).

In our analyses of household consumption and self-perceived health, we found a significant association only between the most deprived group and self-perceived health in the poor vs good model and this relationship was slightly mediated by health behavior variables. The most deprived respondents and women more likely reported their health as poor than the most affluent group. Our findings are in accordance with the results of two recent Serbian studies, which showed that respondents at the lowest level of the socioeconomic distribution, ie, those who belong to the most deprived group had greater morbidity (20) and higher prevalence of chronic diseases (19).

Across European countries, people with lower socioeconomic positions reported worse health than those with higher positions (14,32). Authors from the neighboring Croatia found that a higher proportion of the citizens in the lowest income quartile reported poor health (27.8%) than of their counterparts in the EU member states which joined the EU before May 2004 (9.2%) or in the EU member states which joined the EU in May 2004 (18.6%) (33). A possible interpretation of these findings is that the most disadvantaged groups have fewer material and social resources with which they can deal with their conditions.

The context in which we explored the extent to which smoking mediates inequalities in self-perceived health is unique, since in Serbia people with higher education are more likely to be smokers (21). In our study, smoking and alcohol consumption were strongly associated with good self-perceived health. Bobak et al (34) noticed that selection bias might be a possible reason for these results. Those with good health smoke and drink while those with poor health do not (34). In the fully adjusted model, association between smoking and poor self-rated health was reversed, ie, smokers were most likely to report their health as poor, while the odds ratio for alcohol consumption was slightly changed, but remained protective of poor health. For both health behavior variables, the association with self perceived health remained highly significant. A recent study (21) reported that although persons with higher education in Serbia were more likely to be smokers, they were also more likely to have quit smoking, suggesting that the relationship between socioeconomic position and smoking is in flux. The impact this will have on inequalities in self-perceived health is an important avenue for future research. Our finding that frequent alcohol drinkers had better self-perceived health than non-drinkers is consistent with findings in Russia. Perlman et al (35) reported that frequent alcohol drinkers compared with occasional drinkers had significantly better self-rated health. Also, moderate consumers had better health than abstainers from alcohol. The explanation for this could be that some of the abstainers may previously have been heavy alcohol consumers who were now too ill to drink (36).

This analysis has several limitations. First, the cross-sectional design makes it difficult to establish the temporal relationship, ie, limits the ability to assess the causality between independent variables and self-perceived health as an outcome. This limitation can be overcome with the use of longitudinal studies. A second methodological issue is with regard to the consistency and accuracy of self-perceived health as a health measure used in this study. More objective outcomes will be needed to address this problem. Also, there is a subjective understanding of health associated with self-perceived health status. One French study (37) identified that the concept of health differs between socioeconomic groups, such that middle class respondents were more likely to view “health as well-being,” while working-class respondents defined it as “absence of illness.” Finally, the number of variables we used to explain inequalities was limited. Additional factors that may mediate associations between socioeconomic position and health include psychosocial (stressors, cognitive perception of stressors, psychosocial resources), community (neighborhood conditions, social capital, individual and community level, trust etc.), other health behavior variables (physical activity, healthy diet, body mass index etc), and early life factors (38). In addition, other measures of socioeconomic position such as income would provide a more complete assessment of the relationship between socioeconomic position and self-perceived health.

Despite these limitations, our study is one of the first in Serbia to provide evidence of socioeconomic inequalities in self-perceived health and of the explanatory role of health behavior variables. A marked educational gradient was found with the prevalence of fair or poor self-perceived health in men and women with low educational attainment. After adjustment for demographic and health behavior variables, the association was still strong, suggesting that further research on the determinants of health inequalities in Serbia is necessary. Our research serves as a benchmark to monitor health inequalities during transition, and might increase awareness among policy makers about the scope of inequalities in health. The reduction of such inequalities through wisely tailored interventions that benefit people’s health should be a target of a national health policy in Serbia.
